# Quantitative cone contrast threshold testing in patients with differing pathophysiological mechanisms causing retinal diseases

**DOI:** 10.1186/s40942-023-00442-3

**Published:** 2023-02-02

**Authors:** Kayla M. White, Itamar Livnat, Caroline R. Frambach, John Doan, Urmi V. Mehta, Clara Yuh, Anton M. Palma, Kimberly A. Jameson, M. Cristina Kenney, Mitul C. Mehta, Chantal J. Boisvert, Wade R. Crow, Andrew W. Browne

**Affiliations:** 1 Department of Ophthalmology, Gavin Herbert Eye Institute, University of California, Irvine, CA 92617 USA; 2 Center for Translational Vision Research, University of California, Irvine, CA 92617 USA; 3 School of Medicine, University of California, Irvine, CA 92617 USA; 4 Department of Biomedical Engineering, University of California, Irvine, CA 92617 USA; 5 Institute for Mathematical Behavioral Sciences, University of California, Irvine, CA 92617 USA; 6 Institute for Clinical and Translational Sciences University, Department of Physical Medicine and Rehabilitation, University of California, Irvine, CA 92617 USA; 7 University of California, Irvine, CA 92617 USA; 8 Western University of Health Sciences, Pomona, CA 91766 USA; 9 Medical College of Wisconsin, Wauwatosa, WI 53226 USA; 10 Duke Eye Center, Department of Ophthalmology, Duke University School of Medicine, Durham, NC 27705 USA; 11 Case Western Reserve University Ophthalmology, 10900 Euclid Ave, Cleveland, OH 44106 USA; 12 Kaiser Permanente Santa Clara Internal Medicine, Santa Clara, CA 95051 USA

**Keywords:** Age-related macular degeneration, Color vision, Cone contrast threshold testing (CCT), Epiretinal membrane, Multiple sclerosis, Optic neuritis, Retinal vein occlusion

## Abstract

**Background:**

Cone contrast threshold testing (CCT) provides quantitative measurements of color and contrast function to reveal changes in vision quality that are not standard endpoints in clinical trials. We utilize CCT to measure visual function in patients with multiple sclerosis (MS), age-related macular degeneration (AMD), epiretinal membrane (ERM), and retinal vein occlusion (RVO).

**Methods:**

Retrospective data was gathered from 237 patients of the Gavin Herbert Eye Institute. Subjects included 17 patients with MS, 45 patients with AMD, 41 patients with ERM, 11 patients with RVO, and 123 healthy controls. Patients underwent the primary measurement outcome, CCT testing, as well as Sloan visual acuity test and spectral domain optical coherence tomography during normal care.

**Results:**

Color and contrast deficits were present in MS patients regardless of history of optic neuritis. AMD with intermediate or worse disease demonstrated reduced CCT scores. All 3 stages of ERM demonstrated cone contrast deficits. Despite restoration of visual acuity, RVO-affected eyes demonstrated poorer CCT performance than unaffected fellow eyes.

**Conclusions:**

CCT demonstrates color and contrast deficits for multiple retinal diseases with differing pathophysiology. Further prospective studies of CCT in other disease states and with larger samples sizes is warranted.

**Supplementary Information:**

The online version contains supplementary material available at 10.1186/s40942-023-00442-3.

## Background

Human trichromatic vision has been predicted to detect 2.3 million colors with discrimination between wavelength differences of as little as 0.25 nm. [1, 2] Despite 50 percent of visual information originating from color-sensitive cones in the fovea, clinical visual function testing is largely limited to visual acuity (VA) which quantifies minimal angle of resolution under maximal contrast (black-on-white) conditions [3]. Visual acuity, the widely used “gold standard” for retinal disease clinical trials, have had extensive techniques developed to mitigate the variability in visual acuity charts test results, including the Early Treatment of Diabetic Retinopathy Study (ETDRS) [4]. However, the contributions of color and contrast to visual function are merely noted qualitatively in patients with ophthalmic disease. Routine color vision deficit (CVD) testing in practice is typically reserved for neuro-ophthalmic disease, orbital compressive pathologies, and hereditary retinopathies [5–8] with both advantages and disadvantages for the various color and contrast assays [3]. The anomaloscope, considered the “gold standard” quantitative matching test for assessing CVD [3, 9], is rarely used because it is laborious, time-consuming, and restricted to quantifying CVD in the red-green axis. Instead, pseudoisochromatic plates are frequently used because they are easy to administer, rapid, and inexpensive; however, they provide low resolution quantitative results [3, 9]. Quantitative arrangement tests like the Farnsworth Munsell 100 [10] and computerized adaptations [11] are lengthy and performance is confounded by external factors including nonverbal intelligence. [12]

**Fig. 1:**
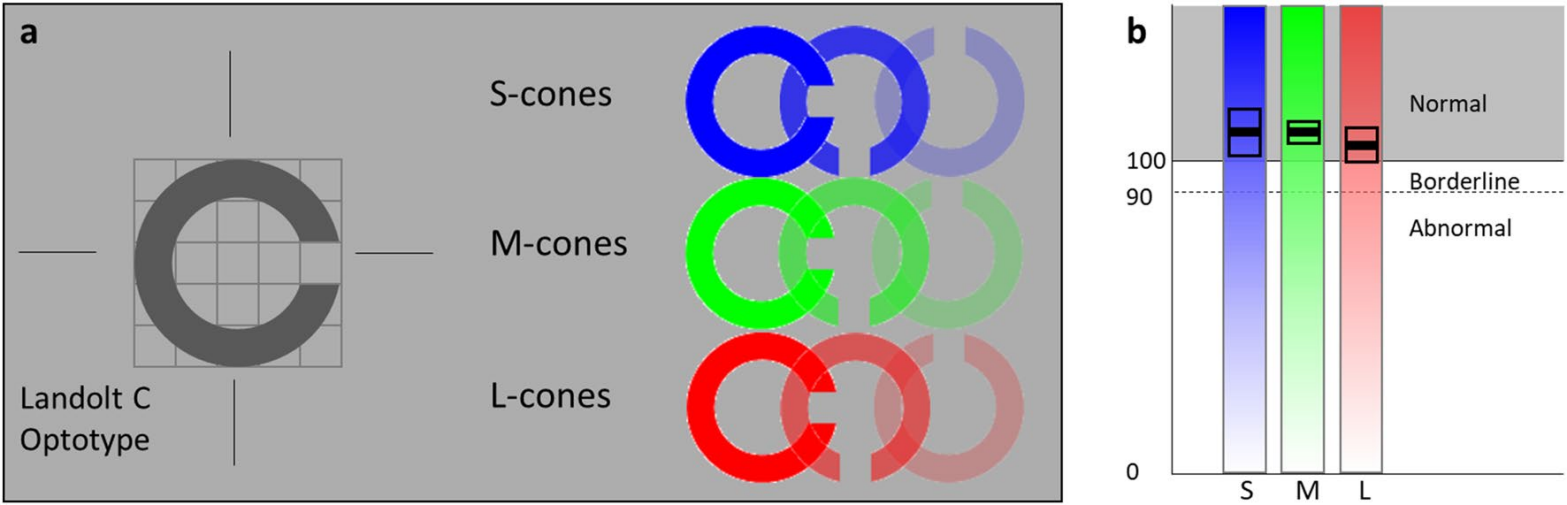
**a** Cone contrast threshold test isolates cone opsins by providing photopic background to suppress rod function and adjusts Landolt C optotype contrast for each cone opsin. **b** The quantitative output indicates a mean score with error for each individual cone opsin and these are reported as normal, borderline or abnormal

The ColorDx Cone Contrast Threshold Test (CCT) (ColorDx HD, Konan Medical, Irvine, CA), a computerized CVD test developed for the US Air Force, uses an adaptive algorithm to consecutively evaluate contrast sensitivity in L-, M-, and S-cones [13]. Individuals are presented with a series of tumbling Landolt-C optotypes in a randomized orientation of cone contrasted against an isochromatic photopic (~ 74 cd/m^2^) background (Fig. 1a) and prompted to indicate the orientation within 5 s. ColorDx generates a continuous quantitative output of contrast sensitivity for each of the 3 cone opsins (Fig. 1b). This test is 4–8 min long, is easy to administer, and can be standardized between testing centers with pre-programed color and luminance calibration on an anti-glare screen. Although not approved by the Food and Drug Administration as a primary endpoint for clinical trials, CCT has demonstrated its use as an indicator of eye function in multiple studies [3, 14]. Military screenings using CCT demonstrate 96–100% sensitivity and specificity for protan, deutan, and tritan CVD compared to the anomaloscope [3, 14]. Our study team has also demonstrated that there was no learning effect, or improvement in scores, when individuals repeated this test multiple times [15].

We report visual function changes beyond visual acuity in patients with 4 diseases affecting the neurosensory retina. Loss of retinal ganglion cells with or without [15] optic neuritis (ON) in multiple sclerosis (MS) has established serial color vision testing as standard care for optic neuropathies [17, 18]. However, color and contrast vision is not standard care in diseases affecting the retina. Age-related macular degeneration (AMD) is a common cause of vision loss primarily due to metabolic dysfunction [19] and death [20] of the photoreceptors and retinal pigmented epithelium, and CCT has been shown to be reduced in the intermediate stage of the disease [21]. Epiretinal membranes (ERM) are thin fibrocellular membranes which exert mechanical force from the inner retina to the outer retina to reduce visual quality and acuity, but little is known about their effect on color and contrast vision [22–24]. Visual acuity loss from retinal edema associated with retinal vein occlusion (RVO), a vascular disease of the inner retina, is well-treated with injectable medications [25–28]; however, many patients still note poor vision despite good visual acuity recovery. In AMD, ERM, and RVO, patients subjectively report qualitative reductions in vision despite normal visual acuity (Additional files 1, 2, Additional file 3: Figs. S1–S3). The aim of this study is to analyze CCT quantitative cone contrast vision testing in patients with MS, AMD, ERM, and RVO to characterize visual function deficits beyond BCVA.

## Methods

### Human subjects

We performed a retrospective chart review on all patients seen at the Gavin Herbert Eye Institute during the period of May 2018 through December 2019 who met inclusion criteria. Institutional Review Board (IRB)/Ethics Committee approval was obtained, and the described research adhered to the tenets of the Declaration of Helsinki. We included patients if they had one of several diagnoses including MS with and without previous ON, AMD, ERM, or RVO. ColorDx is not a visual acuity test but requires 20/120 vision or better to complete the test. All subjects across the various disease states and in the healthy control groups had to meet the following criteria: age 18 or older, no history of other ocular comorbidities except for age-related cataracts, VA of 20/60 or better, and no prior history of ocular surgery except for cataract extraction. To avoid any concern that VA would play any role in a subject’s CCT performance, we elected to conservatively double the minimal acuity for resolution in our population. MS patients were compared to a group of healthy control subjects who met the same inclusion and exclusion criteria as stated above and have no other ocular conditions apart from age-related cataracts and refractive error. A unique group was the set of RVO patients. Given that this disease state affects only one eye in each of our included patients, healthy fellow eyes were compared to disease-impacted eyes within this population to eliminate potential confounding variables between participants. Additionally, in the RVO group, inclusion criteria were broadened in this group to 20/100 or better, which allowed for a larger number of participants while still meeting the 20/120 criteria required by the CCT test. All subjects had previously obtained CCT and VA assessments (Snellen letter set, Sloan characters with ETDRS spacing as is customary on M&S Technologies digital visual acuity charts) as a part of routine care at the Gavin Herbert Eye Institute.

Retina specialists made the diagnosis of AMD, ERM, and RVO (AB, MM). Neurologists in consultation with neuro-ophthalmologists made the diagnosis of MS (CB, WC). Specialists performed dilated retinal exam and optical coherence tomography (OCT) to diagnose and grade AMD by evaluating the extent of pigmentary changes, drusen number, and drusen size [29, 30]. Dilated retinal exam and SD-OCT imaging confirmed the presence of ERM. Two independent retina specialists determined severity and grading of ERM (AB, DF), with a third retina specialist available in the case there was any disagreement between graders [31]. In brief, Grade 1 ERMs have a foveal depres­sion, are thin, and distinguishable retinal layers. Grade 2 membranes have a disruption in their foveal depression, the retina is distorted with widening of layers in particular the outer nuclear layers, however individual layers can still be distinguished. Grade 3 membranes have new continuous ectopic inner foveal layers, but retinal layers can still be distinguished. Grade 4 membranes were not included in this study. Patients were excluded if they were actively receiving treatment for AMD, defined as having received any injections of steroid or anti-VegF within 3 months of performing the CCT assessment. Eyes affected by ERM were compared to a subset of fellow healthy eyes with trace or no ERM and drusen and belonging to patients aged 50 years or older. Eyes affected by AMD were compared to a subset of healthy eyes with no sign of early AMD belonging to patients aged 64 years or older. Age ranges for healthy control groups were restricted to correspond with the age distributions of patients in the diseased groups to minimize age as a possible confounding factor of the study. Diagnosis of RVO was based on clinical examination and a historical onset of more than 6 months and no active macular edema after treatment.

### Cone contrast threshold and imaging

ColorDx is a CCT test that presents a Landolt C optotype in 4 different orientations in the center of a photopic gray screen. Patients use a response pad with arrows to indicate optotype orientation. Following color and luminance calibration on an anti-glare screen, the device presented tumbling Landolt-C optotypes. A Bayesian thresholding method, the Psi-Marginal Adaptive Technique, adjusted the contrast for subsequent tests based on preceding answers [13] and a Psi-marginal calculation yielded the final CCT value. Scores ranged from 175 to 0, with scores below 75 considered as color deficiency in accordance to pass/fail criteria developed by the United States Air Force [3]. The test is performed separately on each eye and generates a separate contrast threshold score for each of the 3 color cones.

The device utilizes an adaptive algorithm per manufacturer specifications to increase or decrease the contrast of the displayed optotype in accordance with the test subject’s response accuracy. All CCT testing was performed with BCVA under photopic and monocular conditions at 2 feet. BCVA, age, and phakic status were recorded for all subjects. SD-OCT (Cirrus, Carl Zeiss Meditiec, USA or Spectralis Heidelberg Engineering Heidelberg Germany) imaging was acquired for all subjects in the disease state groups. The images acquired were horizontal raster scans on either a Zeiss Cirrus 5000 (Karl Zeiss Meditec, Dublin CA) or a Heidelberg Spectralis (Heidelberg Engineering, Heidelberg) through the fovea extending throughout the perifovea. In the MS patient group, OCT images of the retinal nerve fiber layer were analyzed as well. Researchers chose to focus analysis on the average RNFL thickness value as well as temporal RNFL region, which is the most commonly affected region in MS [46]. Additionally, prior literature has demonstrated that thinning of the temporal region has the closest association with disease burden in MS and it is increasingly being utilized to monitor disease state in MS patients with optic neuritis [47, 48].

### Statistical analysis

As each patient’s eyes were subject to different retinal conditions, each eye was considered a separate unit of analysis. Sample characteristics (mean, standard deviation, and range) with respect to age and visual acuity (logMAR) were calculated for all eyes across groups. We then performed linear regression models to estimate the mean differences in CCT scores for each cone class by ERM grade, AMD severity, and, among MS patients, by diagnosis of active optic neuritis and total RNFL thickness (G) or temporal RNFL octant thickness (T) each compared to healthy controls as the reference group. To assess RVO status, we used linear regression models to compare affected vs unaffected eye in each patient. All models were estimated using generalized estimating equations (GEE) to account for clustering within persons and adjusted for age, visual acuity and phakia. For each analysis, individual CCT score distributions for each cone class were visually compared using violin plots with overlying mean CCT score differences with 95% confidence intervals (CIs) for each group. Comparisons for significance were made with healthy controls using adjusted regression model estimates with Pearson’s r correlation coefficients (Additional file 4: Table S1). We conducted all analyses using R version 1.2 [32].

## Results

**Fig. 2:**
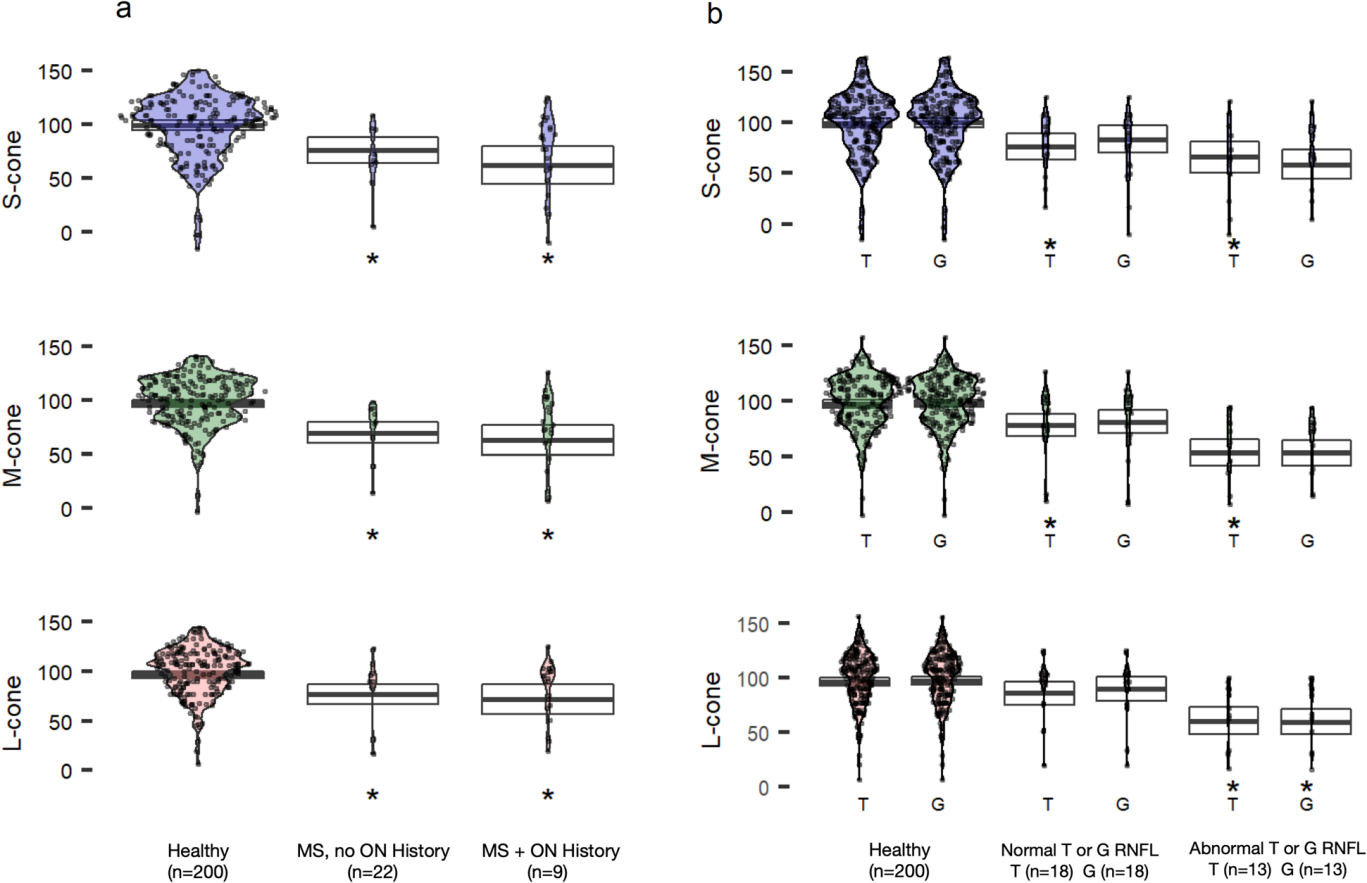
CCT in patients with MS compared to healthy controls. **a** CCT scores in eyes with and without a history of optic neuritis. **b** CCT scores in patients with RNFL thinning in the temporal (T) octant, or the average (G) of all octants. ON = Optic neuritis, RNFL = retinal nerve fiber layer. Estimated means (95% CI) are adjusted for age, visual acuity and phakic status. Asterisks indicate significant differences (vs healthy) at p < 0.05

**Fig. 3:**
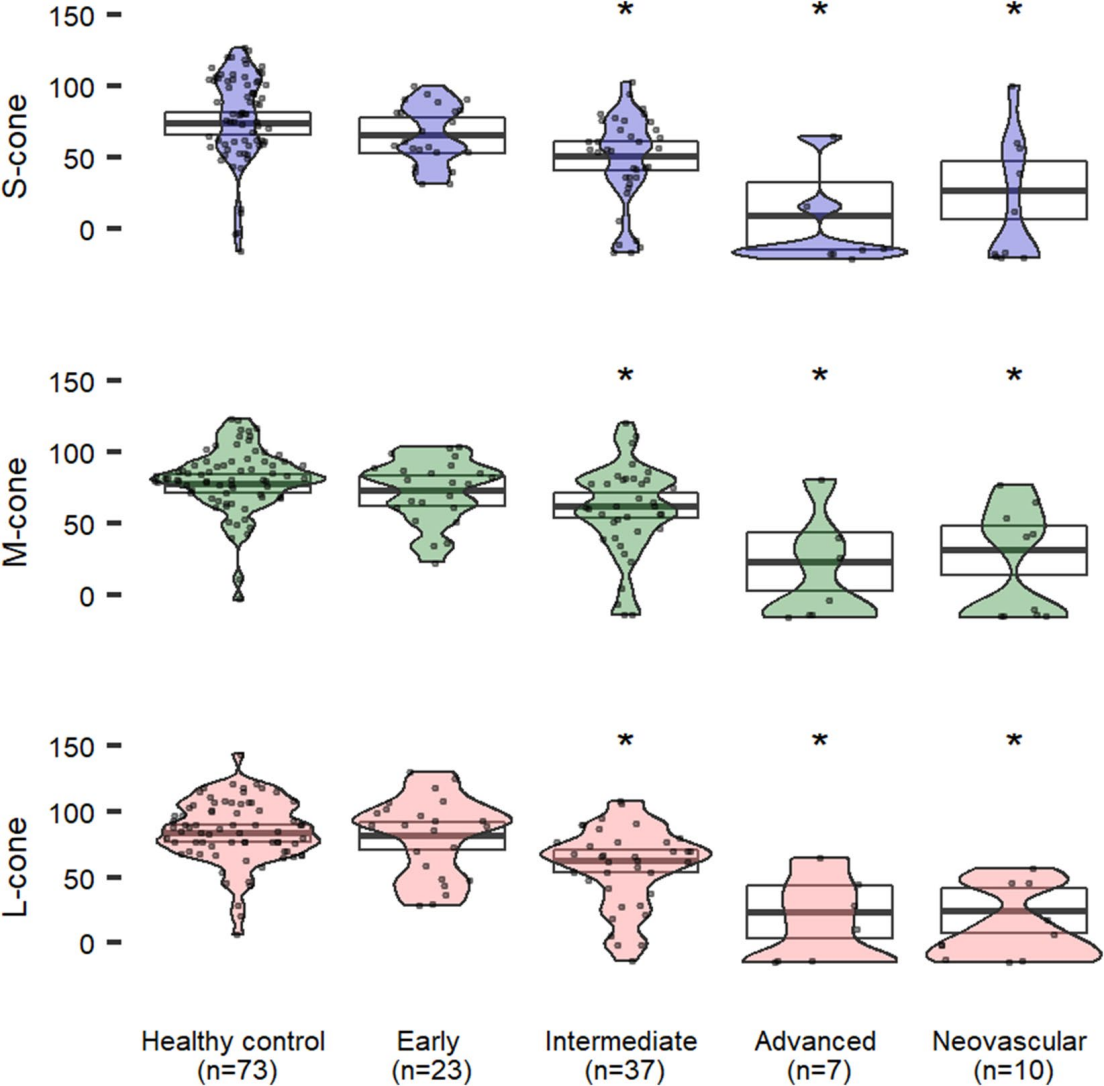
CCT scores in patients with age-related macular degeneration at different stages compared to healthy controls ≥ 64 years old. Estimated means (95% CI) are adjusted for age, visual acuity and phakic status. Asterisks indicate significant differences (versus healthy) at p < 0.05

**Fig. 4:**
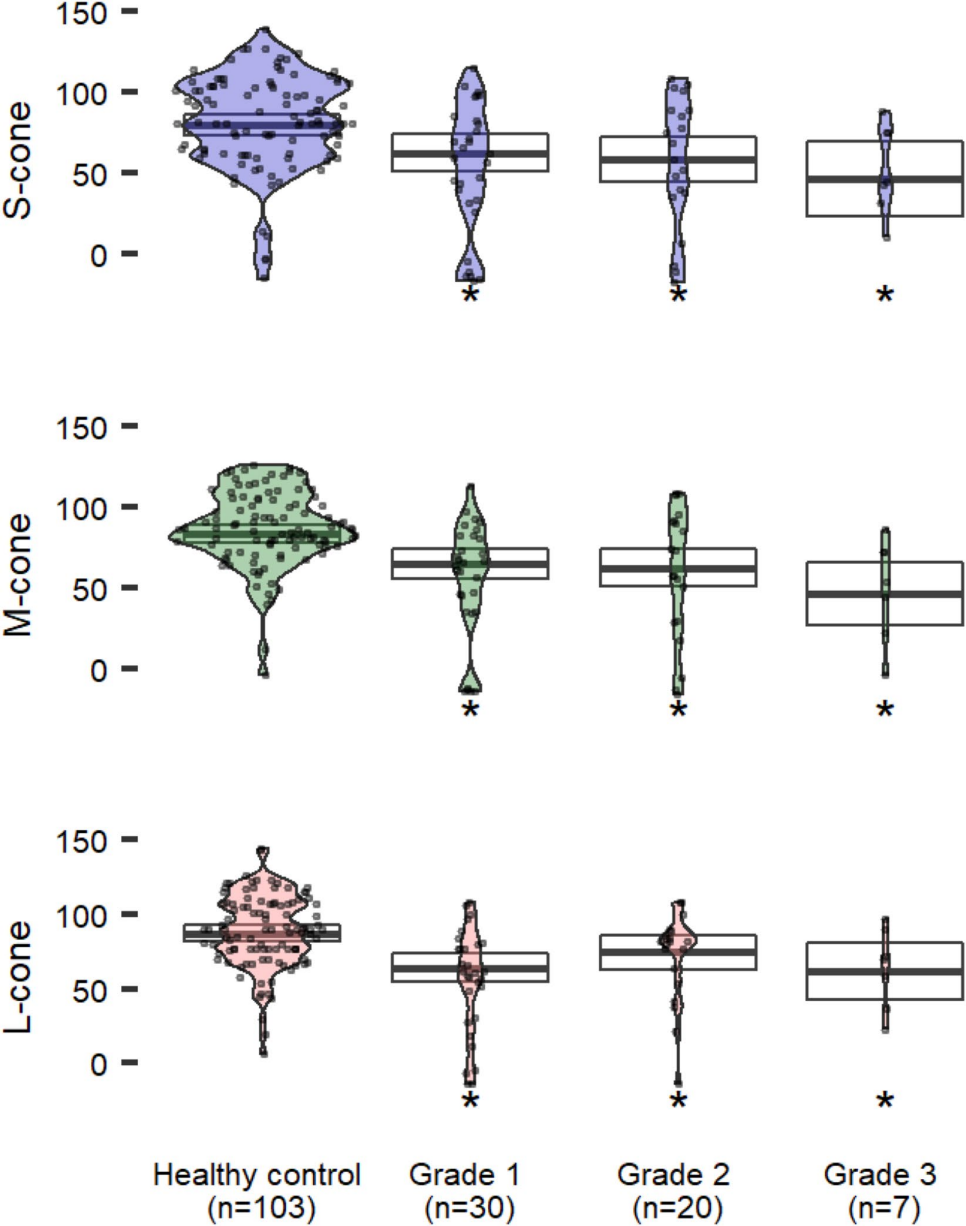
CCT scores in patients with epiretinal membranes increasing in severity compared to healthy controls ≥ 50 years old. Estimated means (95% CI) are adjusted for age, visual acuity and phakic status. Asterisks indicate significant differences (versus healthy) at p < 0.05

**Fig. 5:**
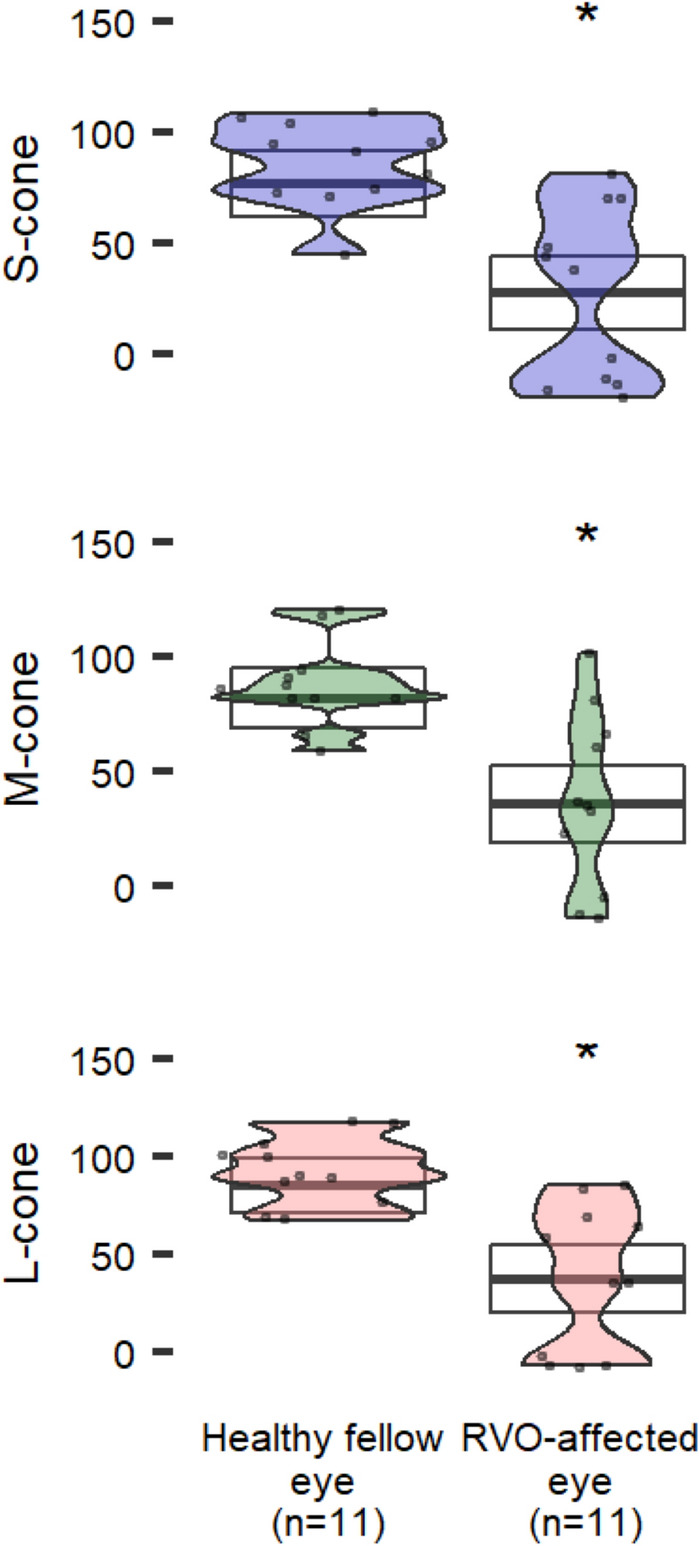
CCT in patients with retinal vein occlusion. Estimated means (95% CI) are adjusted for age, visual acuity and phakic status. Asterisks indicate significant differences (vs healthy) at p < 0.05

Table 1 summarizes the distribution of age and visual acuity of all subjects. CCT results are represented in violin plots that follow (Figs. 2, 3, 4, 5). Each violin plot is a distribution of patients on the horizontal axis with the group CCT scores plotted from 0 to 150 on the vertical axis. The adjusted mean and 95% confidence interval for each plot are overlaid. Significant differences from healthy controls are represented by asterisks.

**Table 1:** Age and visual acuity demographics for healthy control eyes and various retinal disease states

	Group	# Patients, # eyes	Age (mean ± std dev (range))	p-value	Visual Acuity in logMAR (mean ± std dev (range))	p-value	Pseudophakic (n (%))	p-value
MS	Healthy controls	123, 200	48.5 ± 21.9 (19–92)		− 0.03 ± 0.14 (− 0.88 to 0.13)		52 (26%)	
	No History of Optic Neuritis	14, 22	51.3 ± 11.9 (29–70)	0.36	− 0.04 ± 0.10 (− 0.30 to 0.00)	0.22	2 (9%)	0.17
	+ History of Optic Neuritis	6, 9	44.1 ± 11.7 (30–61)	0.31	− 0.09 ± 0.16 (− 0.63 to 0.00)	0.97	0 (0%)	0.14
AMD	Healthy controls (≥ 64 years)	54, 73	72.5 ± 6.0 (64–92)		− 0.09 ± 0.17 (− 0.88 to 0.13)		45 (62%)	
	Early	14, 23	75.6 ± 8.6 (64–94)	.12	− 0.12 ± 0.12 (− 0.40 to 0.00)	0.97	13 (57%)	0.85
	Intermediate	24, 37	79.1 ± 7.6 (67–93)	< .01*	− 0.17 ± 0.16 (− 0.48 to 0.00)	0.19	24 (65%)	0.90
	Advanced	6, 7	82.9 ± 8.7 (69–90)	0.02*	− 0.31 ± 0.20 (− 0.60 to 0.10)	0.08	4 (57%)	0.99
	Neovascular	7, 10	83.5 ± 6.3 (74–93)	< .01*	− 0.30 ± 0.14 (− 0.48 to 0.10)	< .01*	8 (80%)	0.43
**ERM**	Healthy controls (≥ 50 years)	74, 103	72.3 ± 10.4 (50–94)		− 0.18 ± 0.15 (− 0.54 to 0.00)		52 (51%)	
	Stage 1	26, 30	74.7 ± 10.8 (56–94)	< .01*	− 0.14 ± 0.14 (− 0.50 to 0.00)	0.33	20 (67%)	0.17
	Stage 2	17, 20	71.0 ± 10.2 (50–87)	0.24	− 0.19 ± 0.17 (− 0.54 to 0.00)	0.08	13 (65%)	0.34
	Stage 3	6, 7	66.0 ± 6.5 (56–74)	0.45	− 0.28 ± 0.13 (− 0.54 to 0.18)	0.05*	4 (57%)	0.99
RVO	Total	11, 11	68.0 ± 10.7 (51–81)	NA	− 0.26 ± 0.16 (− 0.48 to 0.00)	NA	5 (46%)	NA

^a^The healthy control group includes healthy fellow eyes to eyes with unilateral disease

^b^Patients with eyes categorized in 2 different groups were included in the patient number for both groups

^*^Asterisks denote t-test and chi-square differences that are statistically significant at p < 0.05

### Multiple sclerosis

Not all patients with MS were able to complete CCT in both eyes as some patients had monocular vision loss which precluded test performance. MS patients with a history (9 eyes) and without a history (22 eyes) of ON demonstrated reduced CCT scores compared to the healthy control subject group. Likewise, MS was associated with statistically significant reductions in CCT scores for all cone classes even among those with normal RNFL, except for L-opsin in which normal RNFL was not associated with a significant reduction in CCT (Fig. 2). There was a downtrend in CCT performance in eyes with abnormal RNFL compared to normal RNFL across all 3 color cones that was not statistically significant. There were no statistically significant correlations between VA and CCT score or VA and RNFL.

### Age-related macular degeneration

All classification stages for AMD, except early AMD, demonstrated significant (p < 0.05) reduction in CCT scores compared to healthy control eyes aged 64 and older (Fig. 3). Advanced AMD with or without neovascularization represents the largest reduction in CCT scores demonstrated by widening of the lowest portions of the violin plots. Additionally, no subjects had normal M-cone and L-cone CCT with rare patients in the neovascular AMD retaining some tritan vision (s-cone plot is taller for neovascular AMD than advanced non-neovascular AMD).

### Epiretinal membranes

Two independent retina specialists classified subjects diagnosed on clinical exam and SD-OCT into ERM grades and we evaluated each group’s CCT results (Fig. 4). Each grade of ERM demonstrated a statistically significant reduction in CCT compared with healthy controls aged 50 and older (p < 0.05). Both S- and M-cones demonstrate a nominal reduction in CCT with advancing ERM grade which is not observed with the L-cone, though these differences by ERM grade are not statistically significant.

### Retinal vein occlusion

All patients with RVO (n = 11) who were evaluated with CCT in this study had achieved steady state resolution of macular edema and had sufficient visual acuity scores to reliably complete CCT testing (ranging 20/25 to 20/100). All eleven patients (86%) had visual acuity of 20/60 or better (twice the minimum angle of resolution to reliably complete CCT testing). There was no significant correlation observed between visual acuity and CCT. RVO demonstrated reduced CCT for all 3 cone populations (Fig. 5). For example, one patient with 20/100 visual acuity had near zero CCT results which were similar to other eyes with 20/25 visual acuity. Another patient with 20/80 visual acuity had diminished, but not zero, CCT scores equivalent to other eyes affected by RVO and 20/25 vision.

## Discussion

We analyzed cone contrast threshold testing of patients with a variety of diseases with differing pathophysiological mechanisms for vision loss. CCT performance has been shown to quantify contrast and color vision. For these various diseases, we sought to determine if CCT reveals diminished visual function beyond VA alone. We started by verifying color vision deficits in a disease for which color vision testing is performed as standard of care. Color vision testing is routinely performed in patients with optic neuropathies and diseases like MS which can present with optic neuritis. We then evaluated CCT testing in retinal diseases for which color and contrast vision is not routinely tested.

Multiple Sclerosis is associated with earlier and more pronounced color and contrast vision dysfunction compared to other eye diseases [33]. Color vision loss in MS was previously thought to occur secondary to ON due to direct damage to the optic nerve and resulting damage to the retinal ganglion cell layer [16]. More recently, retinal fiber nerve layer atrophy and reduced macular volume have been described in MS patients with no history of ON [16]. One theory suggests a secondary retinopathy in MS due to retrograde axonal degeneration [17].

Our study demonstrated that MS with no history of ON exhibited a statistically significant reduction in CCT compared to controls (Fig. 2a). This supports the theory that color vision loss occurs as a result of a separate disease process from optic nerve inflammation. The downtrend in CCT scores with abnormal RNFL compared to normal RNFL suggests a correlation between functional vision loss and RNFL, although a larger sample size is needed to further verify this relationship as it did not reach statistical significance (Fig. 2b).

The temporal octant of the optic nerve transmits visual signals from the fovea and perifovea. Thinning of the temporal octant is associated with a significant reduction in CCT scores, and the same is true for the global nerve fiber layer thickness in the optic nerve. Even after adjusting for visual acuity, visual quality subjectively declines, which is corroborated by a decline in CCT performance. Because global and temporal changes in RNFL thickness correlate with CCT reduction, but only the temporal RNFL serves CCT performance, changes in analogous visual quality features may continue in the eccentric and peripheral vision.

Age-related macular degeneration is characterized by the physical presence of drusen with an associated disruption in normal retinal anatomy, physiology, and vision [34]. Early AMD is functionally indistinguishable from normal aging [21]; however, intermediate disease produces reduced color and contrast sensitivity with poor low-light vision which can precede the onset of visual acuity loss. Advanced AMD extinguishes central visual acuity and contrast vision. Clinical trials directed at treating or slowing progression of diseases like non-neovascular (dry) AMD rely exclusively on subjective VA determinations and objective structural imaging [35]. Some new treatments show promise because they slow structural damage [36] while other potential treatments are discarded after multimillion dollar clinical trials because they do not show the same structural benefits [37]. While retinal structure and visual acuity do correlate, subjective measures of visual function extend beyond visual acuity to contrast sensitivity, color perception, and the ability to respond to changing lighting conditions (Additional file 1: Fig. S1). These other subjective visual function tests are not routinely performed because they are slow, require trained technical administration, demand reliable patient participation, and do not integrate easily with a busy clinic schedule.

AMD patients described in this report confirm similar findings to Cocce et. al [23]. with CCT results insignificantly changed in early AMD, but intermediate and advanced AMD yield large reductions in color vision. There is partial agreement with the data presented by Oza et. al [38]. which found significant CCT changes in all 3 color vision tests in intermediate AMD; they also found changes in early AMD when tracking patients over the course of 12 and 24 months, which was not in the scope of the current study. CCT may be used as a clinical endpoint for interventions directed at intermediate AMD and beyond. Inability for conventional testing to identify functional endpoints for early AMD highlights our communities need for novel assays sensitive to early disease.

Epiretinal membranes are thin avascular fibrocellular membranes which develop on the inner surface of the retina [22, 39]. ERMs can be asymptomatic or alter macular structure to produce metamorphopsia and reduced visual acuity [22–24]. SD-OCT studies of the ERM reveal retinal disruptions that may impair cone function such as blurred, interrupted, or absent cone outer segment tip lines (COST) [21]. Additionally, the ectopic inner foveal layers (EIFLs) within ERMs have been associated with significant visual function loss [31]. Disruptions of the COST line has been associated with macular diseases and may indicate photoreceptor dysfunction [40]. Recovery of the COST after ERM surgery correlates with better outcomes in VA, with COST thickness prior to ERM surgery correlating well to postoperative VA [41, 42]. While some patients subjectively report metamorphopsia and reduced acuity, others additionally report reduced quality in their vision (Additional file 2: Fig. S2). However, little is known about the qualitative changes in vision beyond visual acuity.

Utilizing CCT, we demonstrate significant color and contrast deficits in patients with ERM’s of mild to severe grade. While not statistically significant, there was a downtrend in CCT performance with worsening ERM grade in S and M cones and further research is warranted to determine if disease severity correlates with the degree of color and contrast vision loss.

Retinal vein occlusion remains one of the most investigated retinal disease conditions because intraocular anti-VEGF and steroid injections demonstrate profound improvements in retinal anatomy seen on OCT and visual acuity [43]. RVO, like diabetic retinopathy, is an inner retinal vascular disease affecting cells in the ganglion cell layer and inner nuclear layer. Clinical trials to treat eyes affected by RVO focus on visual acuity and OCT anatomy as the primary outcome for therapeutic success. However, patients who have recovered visual acuity suitable for reading or driving remain dissatisfied with the quality of their vision (Additional file 3: Fig. S3).

CCT testing in patients who have recovered optimal visual acuity and retinal anatomy highlights the residual loss of color and contrast vision in eyes affected by RVO when compared with unaffected fellow eyes. This finding was present for both eyes with 20/40 vision or better (n = 8), and eyes with vision worse than 20/40 (n = 6). CCT, therefore, presents a practical quantitative endpoint for therapies aimed at optimizing visual quality in addition to visual acuity. These results agree with Matsumoto et. al. [44], who demonstrated similar findings in statistically significant loss of all S, M, and L cones testing in eyes affected by RVO when compared to the healthy eye.

Overall, the data presented in this report demonstrates an agreement with previous studies that there are various disease states associated with reduced CCT scores. Improvement of S and M scores were seen after cataract surgery, suggesting worsening S and M CCT scores with cataract development.15 Worsened M and S CCT scores have been demonstrated in glaucoma. Our data demonstrated MS was associated with reductions in CCT scores, except for L-opsin in which MS patients with normal RNFL did not have significant reduction in CCT. Prior studies on AMD agreed with the current data that there are reductions in CCT scores across all cones in intermediate AMD and advanced AMD [23, 38]. We found no statistically significant changes in early AMD, which partially disagrees with Oza et. al [38]. who found changes in early AMD when tracking patients over time. Regarding ERM, our data demonstrated statistically significant worsened CCT scores across S, M, and L cones compared to healthy controls, however there was no statistically significant differences in scores across worsening ERM grades. Finally, we found worsened S, M, and L cone CCT scores in eyes affected by RVO compared to unaffected fellow eyes, which agrees with published studies [44].

Results of this study are likely impacted by its retrospective nature, and limited sample size, particularly for the higher grade ERM states. ETDRS testing was not used because all data was collected during standard course of care where the M&S technologies digital display for visual acuity testing. Additionally, it is worth noting that while healthy control eyes were restricted to age ranges of the corresponding AMD and ERM disease state eyes, there were statistically significant increases in the ages for intermediate, advanced, and neovascular AMD as well as trace ERM overall when compared to their healthy control groups. While this report highlights changes in color and contrast vision with disease, further prospective studies with larger sample sizes are warranted.

## Conclusion

As one of the first studies to utilize CCT testing in MS, AMD, ERM, and RVO patients, we present novel quantitative data describing cone-specific visual function across all 3 cone classes in these disease states. We observed that inner retinal vascular and inner retinal mechanical disease states both diminish CCT test results, suggesting that CCT performance is affected by multiple mechanisms downstream of photoreceptors.

## Supplementary Information

**Additional file 1: Fig. S1.** 94-year-old pseudophakic male with quiescent nvAMD in his right eye and non-nvAMD in his left eye. The patient reports that color blocks in the CCT report appear black in his right eye (VA: 20/40), and washed out in his 20/20 left eye (VA: 20/20).

**Additional file 2: Fig. S2.** 72-year-old pseudophakic female reports visual distortion and 20/40 vision OD with normal 20/20 vision OS. SD-OCT imaging of right eye demonstrates ERM with corresponding reduction in CCT. *Orange line denotes cut-off point for normal cone contrast scores.

**Additional file 3: Fig. S3**. a) Fundus Autofluorescence images and visual acuities for 65-year-old phakic female who presented with acute onset blurry vision OD was found to have an inferior hemiretinal RVO and cystoid macular edema. b) After anti-VEGF therapy the patient’s vision improved from 20/60 to 20/30 but she remained very symptomatic for “poor” vision. CCT scores OD were significantly lower and near zero OD compared to OS even when visual acuity was 20/30 and 20/15.

**Additional file 4: Table S1**. Regression model beta coefficients, 95% confidence intervals (CIs) and p-values for model predictors.

## Data Availability

The datasets generated and/or analyzed during the current study are available from the corresponding author on reasonable request.

## References

[1] Pointer MR, Attridge GG. The number of discernible colours. Color Research & Application: Endorsed by Inter‐Society Color Council, The Colour Group (Great Britain), Canadian Society for Color, Color Science Association of Japan, Dutch Society for the Study of Color, The Swedish Colour Centre Foundation, Colour Society of Australia, Centre Français de la Couleur 23.1 (1998): 52–54.

[2] Mollon JD, Estévez O, Cavonius CR. The two subsystems of colour vision and their roles in wavelength discrimination. In: Vision: coding and efficiency. 1990; p. 119–131.

[3] Walsh DV, Robinson J, Jurek GM, Capo-Aponte JE, Riggs DW, Temme LA (2016). A performance comparison of color vision tests for military screening. Aerosp Med Hum Perform.

[4] Kaiser PK (2009). Prospective evaluation of visual acuity assessment: a comparison of snellen versus ETDRS charts in clinical practice (An AOS Thesis). Trans Am Ophthalmol Soc.

[5] Flanagan P, Zele AJ (2004). Chromatic and luminance losses with multiple sclerosis and optic neuritis measured using dynamic random luminance contrast noise. Ophthalmic Physiol Opt.

[6] Hudson G, Yu-Wai-Man P, Chinnery PF (2008). Leber hereditary optic neuropathy. Expert Opin Med Diagn.

[7] Moura ALDA, Teixeira RAA, Oiwa NN, Costa MF, Feitosa-Santana C, Callegaro D, Hamer RD, Ventura DF (2008). Chromatic discrimination losses in multiple sclerosis patients with and without optic neuritis using the Cambridge Colour Test. Vis Neurosci.

[8] Rajabi MT, Ojani M, RiaziEsfahani H, Tabatabaei SZ, Rajabi MB, Hosseini SS (2019). Correlation of peripapillary nerve fiber layer thickness with visual outcomes after decompression surgery in subclinical and clinical thyroid-related compressive optic neuropathy. J Curr Ophthalmol.

[9] FanloZarazaga A, Gutierrez Vasquez J, Pueyo RV (2019). Review of the main colour vision clinical assessment tests. Arch Soc Esp Oftalmol.

[10] Birch J (1989). Use of the Farnsworth-Munsell 100-Hue test in the examination of congenital colour vision defects. Ophthalmic Physiol Opt.

[11] Ghose S, Parmar T, Dada T, Vanathi M, Sharma S (2014). A new computer-based Farnsworth Munsell 100-hue test for evaluation of color vision. Int Ophthalmol.

[12] Cranwell MB, Pearce B, Loveridge C, Hurlbert AC (2015). Performance on the Farnsworth-Munsell 100-Hue test is significantly related to nonverbal IQ. Invest Ophthalmol Vis Sci.

[13] Prins N (2013). The psi-marginal adaptive method: how to give nuisance parameters the attention they deserve (no more, no less). J Vis.

[14] Rabin J, Gooch J, Ivan D (2011). Rapid quantification of color vision: the cone contrast test. Invest Ophthalmol Vis Sci.

[15] Mehta U, Diep A, Nguyen K, Le B, Yuh C, Frambach C, Doan J, Wei A, Palma AM, Farid M, Garg S (2020). Quantifying color vision changes associated with cataracts using cone contrast thresholds. Tran Vis Sci Tech..

[16] Lampert EJ, Andorra M, Torres-Torres R, Ortiz-Pérez S, Llufriu S, Sepúlveda M, Sola N, Saiz A, Sánchez-Dalmau B, Villoslada P, Martínez-Lapiscina EH (2015). Color vision impairment in multiple sclerosis points to retinal ganglion cell damage. J Neurol.

[17] Felgueiras H, Parra J, Cruz S, Pereira P, Santos AF, Rua A, Meira D, Fonseca P, Pedrosa C, Cardoso JN, Almeida C (2017). Dyschromatopsia in multiple sclerosis patients: a marker of subclinical involvement?: Response. J Neuroophthalmol.

[18] Martínez-Lapiscina EH, Ortiz-Pérez S, Fraga-Pumar E, Martínez-Heras E, Gabilondo I, Llufriu S, Bullich S, Figueras M, Saiz A, Sánchez-Dalmau B, Villoslada P (2014). Colour vision impairment is associated with disease severity in multiple sclerosis. Mult Scler.

[19] Al-Zamil WM, Yassin SA (2017). Recent developments in age-related macular degeneration: a review. Clin Interv Aging.

[20] Gheorghe A, Mahdi L, Musat O (2015). Age-related macular degeneration. Rom J Ophthalmol.

[21] Cocce KJ, Stinnett SS, Luhmann UF, Vajzovic L, Horne A, Schuman SG, Toth CA, Cousins SW, Lad EM (2018). Visual function metrics in early and intermediate dry age-related macular degeneration for use as clinical trial endpoints. Am J Ophthalmol.

[22] Bu SC, Kuijer R, Li XR, Hooymans JM, Los LI (2014). Idiopathic epiretinal membrane. Retina.

[23] Watanabe K, Tsunoda K, Mizuno Y, Akiyama K, Noda T (2013). Outer retinal morphology and visual function in patients with idiopathic epiretinal membrane. JAMA Ophthalmol.

[24] Koo HC, Rhim WI, Lee EK (2012). Morphologic and functional association of retinal layers beneath the epiretinal membrane with spectral-domain optical coherence tomography in eyes without photoreceptor abnormality. Graefes Arch Clin Exp Ophthalmol.

[25] Varma R, Bressler NM, Suñer I, Lee P, Dolan CM, Ward J, Colman S, Rubio RG (2012). Improved vision-related function after ranibizumab for macular edema after retinal vein occlusion: results from the BRAVO and CRUISE trials. Ophthalmology.

[26] Brown DM, Heier JS, Clark WL, Boyer DS, Vitti R, Berliner AJ, Zeitz O, Sandbrink R, Zhu X, Haller JA (2013). Intravitreal aflibercept injection for macular edema secondary to central retinal vein occlusion: 1-year results from the phase 3 COPERNICUS study. Am J Ophthalmol..

[27] Ip MS, Scott IU, VanVeldhuisen PC, Oden NL, Blodi BA, Fisher M, Chan CK, Gonzalez VH, Singerman LJ, Tolentino M (2009). A randomized trial comparing the efficacy and safety of intravitreal triamcinolone with observation to treat vision loss associated with macular edema secondary to central retinal vein occlusion: the Standard Care vs Corticosteroid for Retinal Vein Occlusion (SCORE) study report 5. Arch Ophthalmol.

[28] Scott IU, Ip MS, VanVeldhuisen PC, Oden NL, Blodi BA, Fisher M, Chan CK, Gonzalez VH, Singerman LJ, Tolentino M (2009). A randomized trial comparing the efficacy and safety of intravitreal triamcinolone with standard care to treat vision loss associated with macular Edema secondary to branch retinal vein occlusion: the Standard Care vs Corticosteroid for Retinal Vein Occlusion (SCORE) study report. Arch Ophthalmol.

[29] Ferris FL, Davis MD, Clemons TE, Lee LY, Chew EY, Lindblad AS, Milton RC, Bressler SB, Klein R (2005). A simplified severity scale for age-related macular degeneration: AREDS Report. Arch Ophthalmol..

[30] Liew G, Joachim N, Mitchell P, Burlutsky G, Wang JJ (2016). Validating the AREDS simplified severity scale of age-related macular degeneration with 5- and 10-year incident data in a population-based sample. Ophthalmology.

[31] Govetto A, Lalane RA, Sarraf D, Figueroa MS, Hubschman JP (2017). Insights into epiretinal membranes: presence of ectopic inner foveal layers and a new optical coherence tomography staging scheme. Am J Ophthalmol.

[32] R Core Team. R: a language and environment for statistical computing. R Foundation for Statistical Computing, Vienna, Austria. http://www.R-project.org/. In:2019.

[33] Almog Y, Nemet A (2010). The correlation between visual acuity and color vision as an indicator of the cause of visual loss. Am J Ophthalmol.

[34] Pennington KL, DeAngelis MM (2016). Epidemiology of age-related macular degeneration (AMD): associations with cardiovascular disease phenotypes and lipid factors. Eye Vis (Lond).

[35] Spaide RF, Curcio CA (2010). Drusen characterization with multimodal imaging. Retina.

[36] Apellis Pharmaceuticals I. Study of of APL-2 Therapy in Patients Geographic Atrophy (FILLY). In. (Clinicaltrials.gov Identifier NCT0250332)2015.

[37] Holz FG, Sadda SR, Busbee B, Chew EY, Mitchell P, Tufail A, Brittain C, Ferrara D, Gray S, Honigberg L, Martin J (2018). Efficacy and safety of lampalizumab for geographic atrophy due to age-related macular degeneration: chroma and spectri phase 3 randomized clinical trials. JAMA Ophthalmol.

[38] Oza VH, Kurzlechner L, Nordstrom C, Luhmann UF, Stinnett S, Lad EM (2022). Cone contrast testing as an early functional biomarker of age-related macular degeneration. Invest Ophthalmol Vis Sci.

[39] Gandorfer A, Rohleder M, Kampik A (2002). Epiretinal pathology of vitreomacular traction syndrome. Br J Ophthalmol.

[40] Tsunoda K, Watanabe K, Akiyama K, Usui T, Noda T (2012). Highly reflective foveal region in optical coherence tomography in eyes with vitreomacular traction or epiretinal membrane. Ophthalmology.

[41] Itoh Y, Inoue M, Rii T, Hirota K, Hirakata A (2013). Correlation between foveal cone outer segment tips line and visual recovery after epiretinal membrane surgery. Invest Ophthalmol Vis Sci.

[42] Rii T, Itoh Y, Inoue M, Hirota K, Hirakata A (2014). Outer retinal morphological changes and visual function after removal of epiretinal membrane. Can J Ophthalmol.

[43] Ho M, Liu DT, Lam DS, Jonas JB (2016). Retinal vein occlusions, from basics to the latest treatment. Retina.

[44] Matsumoto R, Saishin Y, Ohji M (2021). Evaluation of acquired color vision deficiency in retinal vein occlusion using the Rabin cone contrast test. Graefe's Arch Clin Exp Ophthalmol.

[45] Niwa Y, Muraki S, Naito F, Minamikawa T, Ohji M (2014). Evaluation of acquired color vision deficiency in glaucoma using the Rabin cone contrast test. Inv Ophthalmol Vis Sci.

[46] Wang XL, Yu T, Xia DEZ, Zhang JS, Yan QIC, Luo YAH (2010). Measurement of retinal nerve fiber layer thickness in optic atrophy eyes of patients with optic neuritis using optical coherence tomography. Graefe’s Arch Clin Exp Ophthalmol.

[47] Birkeldh U, Manouchehrinia A, Hietala MA, Hillert J, Olsson T, Piehl F, Kockum IS, Brundin L, Zahavi O, Wahlberg-Ramsay M, Brautaset R, Nilsson M (2017). The temporal retinal nerve fiber layer thickness is the most important optical coherence tomography estimate in multiple sclerosis. Front Neurol.

[48] Bertuzzi F, Suzani M, Tagliabue E, Cavaletti G, Angeli R, Balgera R, Rulli E, Ferrarese C, Miglior S (2010). Diagnostic validity of optic disc and retinal nerve fiber layer evaluations in detecting structural changes after optic neuritis. Ophthalmology.

