# Supplementary material for: Quantitative cone contrast threshold testing in patients with differing pathophysiological mechanisms causing retinal diseases
Source: Int J Retina Vitreous. 2023 Feb 2;9:9. doi: 10.1186/s40942-023-00442-3 (PMC9893567; doi:10.1186/s40942-023-00442-3)
Supplement: Supplementary file 1 — Additional file 1: Fig. S1. 94-year-old pseudophakic male with quiescent nvAMD in his right eye and non-nvAMD in his left eye. The patient reports that color blocks in the CCT report appear black in his right eye (VA: 20/40), and washed out in his 20/20 left eye (VA: 20/20). [file 40942_2023_442_MOESM1_ESM.docx]

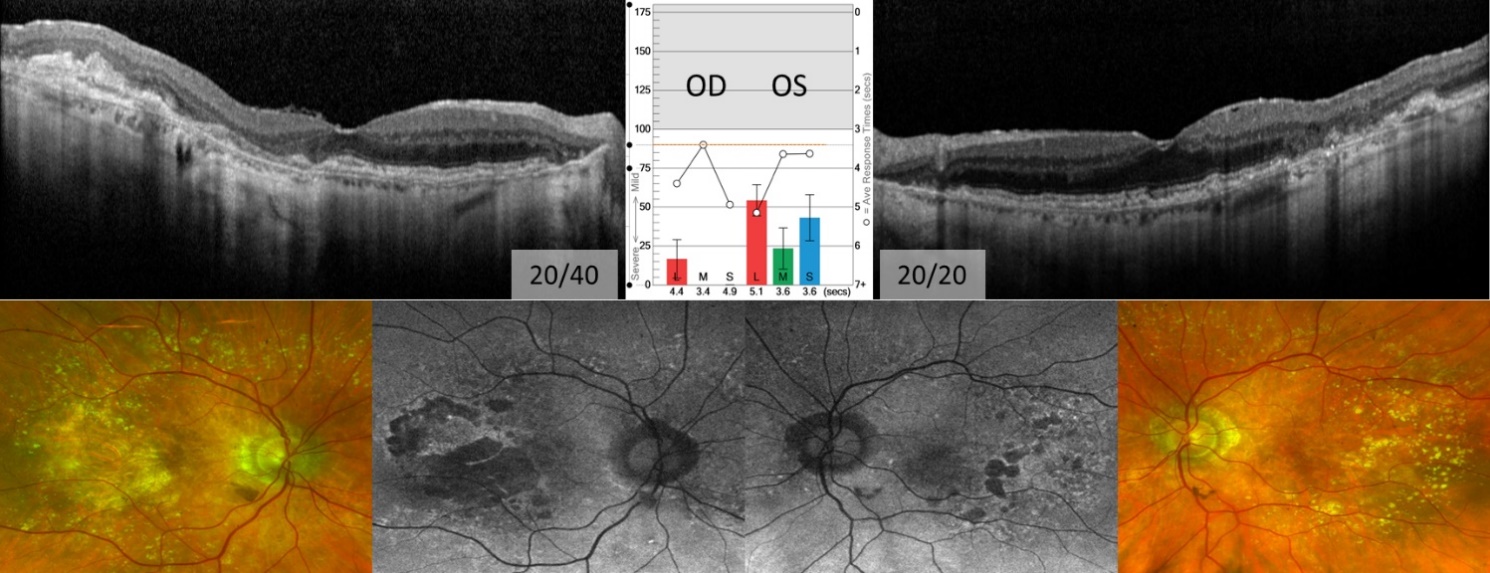
 OD OS

**Figure S1:** 94-year-old pseudophakic male with quiescent nvAMD in his right eye and non-nvAMD in his left eye. The patient reports that color blocks in the CCT report appear black in his right eye (VA: 20/40), and washed out in his 20/20 left eye (VA: 20/20).
