# Supplementary material for: Quantitative cone contrast threshold testing in patients with differing pathophysiological mechanisms causing retinal diseases
Source: Int J Retina Vitreous. 2023 Feb 2;9:9. doi: 10.1186/s40942-023-00442-3 (PMC9893567; doi:10.1186/s40942-023-00442-3)
Supplement: Supplementary file 2 — Additional file 2: Fig. S2. 72-year-old pseudophakic female reports visual distortion and 20/40 vision OD with normal 20/20 vision OS. SD-OCT imaging of right eye demonstrates ERM with corresponding reduction in CCT. *Orange line denotes cut-off point for normal cone contrast scores. [file 40942_2023_442_MOESM2_ESM.docx]

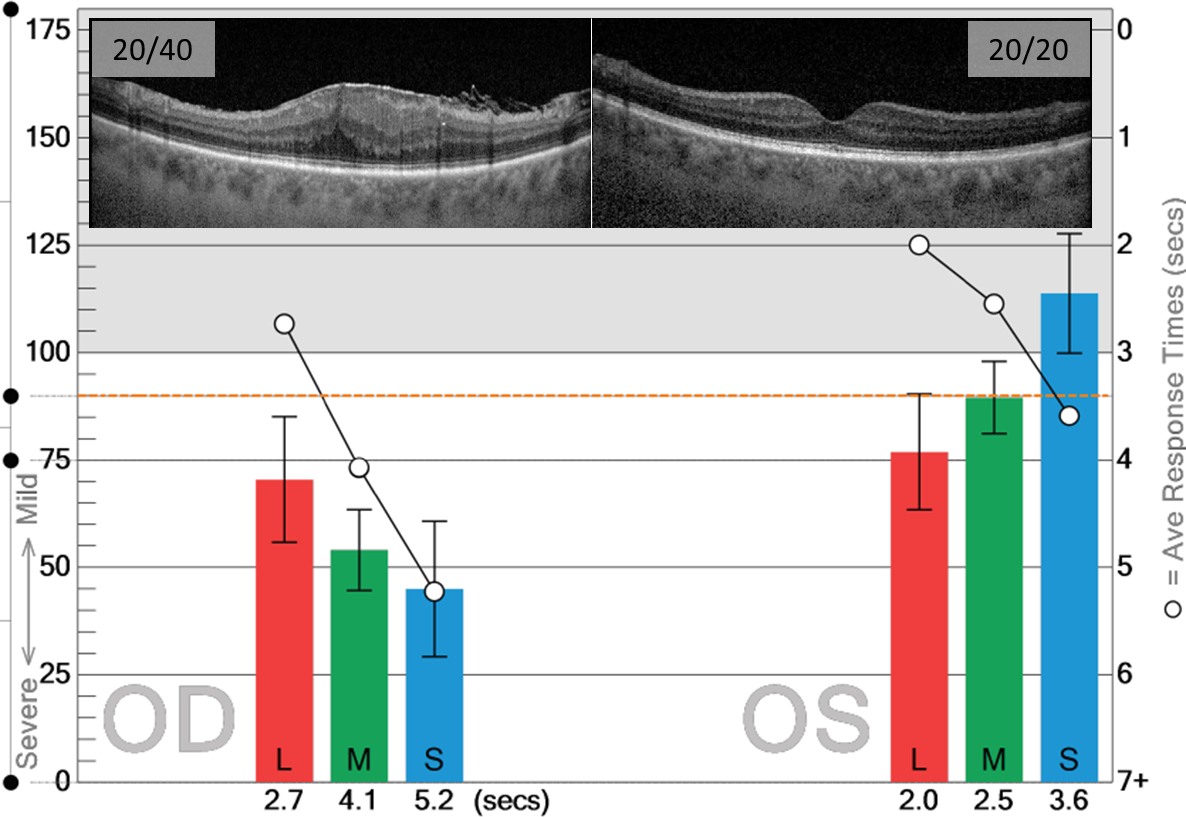


**Figure S2:** 72-year-old pseudophakic female reports visual distortion and 20/40 vision OD with normal 20/20 vision OS. SD-OCT imaging of right eye demonstrates ERM with corresponding reduction in CCT. *Orange line denotes cut-off point for normal cone contrast scores.
