# Supplementary material for: Quantitative cone contrast threshold testing in patients with differing pathophysiological mechanisms causing retinal diseases
Source: Int J Retina Vitreous. 2023 Feb 2;9:9. doi: 10.1186/s40942-023-00442-3 (PMC9893567; doi:10.1186/s40942-023-00442-3)
Supplement: Supplementary file 3 — Additional file 3: Fig. S3. a) Fundus Autofluorescence images and visual acuities for 65-year-old phakic female who presented with acute onset blurry vision OD was found to have an inferior hemiretinal RVO and cystoid macular edema. b) After anti-VEGF therapy the patient’s vision improved from 20/60 to 20/30 but she remained very symptomatic for “poor” vision. CCT scores OD were significantly lower and near zero OD compared to OS even when visual acuity was 20/30 and 20/15. [file 40942_2023_442_MOESM3_ESM.docx]

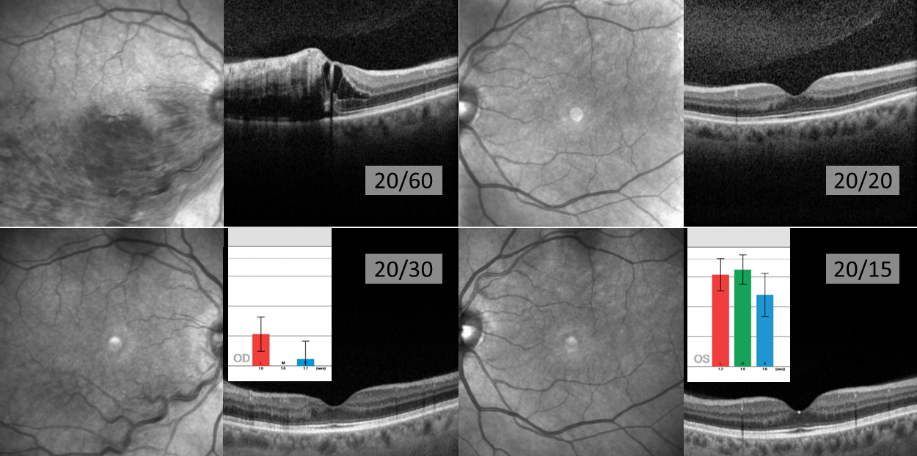


Figure S3: a) Fundus Autofluorescence images and visual acuities for 65-year-old phakic female who presented with acute onset blurry vision OD was found to have an inferior hemiretinal RVO and cystoid macular edema. b) After anti-VEGF therapy the patient’s vision improved from 20/60 to 20/30 but she remained very symptomatic for “poor” vision. CCT scores OD were significantly lower and near zero OD compared to OS even when visual acuity was 20/30 and 20/15
