# Supplementary material for: Quantitative cone contrast threshold testing in patients with differing pathophysiological mechanisms causing retinal diseases
Source: Int J Retina Vitreous. 2023 Feb 2;9:9. doi: 10.1186/s40942-023-00442-3 (PMC9893567; doi:10.1186/s40942-023-00442-3)
Supplement: Supplementary file 4 — Additional file 4: Table S1. Regression model beta coefficients, 95% confidence intervals (CIs) and p-values for model predictors. [file 40942_2023_442_MOESM4_ESM.docx]

| Table S1. Regression model beta coefficients, 95% confidence intervals (CIs) and p-values for model predictors. | | | | | | | | | |
| --- | --- | --- | --- | --- | --- | --- | --- | --- | --- |
|  | **S-cone** | | | **M-cone** | | | **L-cone** | | |
| **Model / parameter** | **Coefficient** | **95% CI** | **p-value** | **Coeff** | **95% CI** | **p-value** | **Coeff** | **95% CI** | **p-value** |
|  |  |  |  |  |  |  |  |  |  |
| **ERM vs. healthy control** |  |  |  |  |  |  |  |  |  |
| ERM grade |  |  |  |  |  |  |  |  |  |
| Healthy control | (reference) |  |  |  |  |  |  |  |  |
| Grade 1 | -17 | (-30.1, -3.9) | 0.012 | -18.4 | (-29.1, -7.7) | 0.001 | -22.9 | (-33.6, -12.2) | 0.000 |
| Grade 2 | -20.6 | (-35.5, -5.6) | 0.008 | -20.8 | (-33.0, -8.6) | 0.001 | -12.8 | (-24.9, -0.6) | 0.042 |
| Grade 3 | -32.2 | (-56.3, -8.2) | 0.009 | -36.7 | (-56.4, -17) | 0.000 | -25.7 | (-45.3, -6.1) | 0.011 |
| Age (centered) | -1.4 | (-2.0, -0.8) | 0.000 | -1.3 | (-1.8, -0.8) | 0.000 | -0.9 | (-1.4, -0.4) | 0.000 |
| logMAR | -0.4 | (-30.9, 30.0) | 0.977 | -11.9 | (-36.9, 13.0) | 0.350 | -2.5 | (-27.4, 22.3) | 0.842 |
| Pseudophakic | -1.1 | (-12.0, 9.8) | 0.842 | -7.7 | (-16.6, 1.2) | 0.092 | -13.1 | (-22.0, -4.3) | 0.004 |
|  |  |  |  |  |  |  |  |  |  |
| **AMD vs. healthy control** |  |  |  |  |  |  |  |  |  |
| AMD severity |  |  |  |  |  |  |  |  |  |
| Healthy control | (reference) |  |  |  |  |  |  |  |  |
| Early | -9.4 | (-23.3, 4.5) | 0.186 | -5.8 | (-17.6, 6) | 0.336 | -2.1 | (-14, 9.9) | 0.736 |
| Intermediate | -23.8 | (-36.4, -11.2) | 0.000 | -15.7 | (-26.4, -5) | 0.005 | -21.1 | (-32, -10.3) | 0.000 |
| Advanced | -66.4 | (-91, -41.8) | 0.000 | -54.4 | (-75.3, -33.5) | 0.000 | -61.1 | (-82.3, -40) | 0.000 |
| Neovascular | -48 | (-69.4, -26.7) | 0.000 | -45.8 | (-63.9, -27.7) | 0.000 | -59.7 | (-78.1, -41.4) | 0.000 |
| Age (centered) | -0.7 | (-1.3, -0.1) | 0.017 | -0.9 | (-1.4, -0.4) | 0.001 | -0.6 | (-1.1, -0.1) | 0.015 |
| logMAR | -23.9 | (-51.9, 4.1) | 0.096 | -19.6 | (-43.4, 4.2) | 0.108 | -13.6 | (-37.6, 10.5) | 0.271 |
| Pseudophakic | -1 | (-10.8, 8.9) | 0.846 | -6.4 | (-14.7, 2) | 0.136 | -14.7 | (-23.2, -6.3) | 0.001 |
|  |  |  |  |  |  |  |  |  |  |
| **RVO-affected vs. healthy fellow eye** |  |  |  |  |  |  |  |  |  |
| RVO status |  |  |  |  |  |  |  |  |  |
| Healthy fellow eye | (reference) |  |  |  |  |  |  |  |  |
| RVO-affected | -29.7 | (-63.3, 3.9) | 0.083 | -31.6 | (-56.8, -6.3) | 0.014 | -32.4 | (-61.3, -3.6) | 0.028 |
| Age (centered) | 0.9 | (-0.9, 2.7) | 0.340 | -0.7 | (-2.5, 1.1) | 0.432 | 0 | (-1.8, 1.7) | 0.982 |
| logMAR | -114.6 | (-237.6, 8.3) | 0.068 | -83.6 | (-198.9, 31.7) | 0.155 | -103 | (-224.7, 18.7) | 0.097 |
| Pseudophakic | -24.5 | (-51.1, 2.1) | 0.071 | -15.1 | (-56.4, 26.1) | 0.473 | -16.4 | (-53.8, 20.9) | 0.388 |
|  |  |  |  |  |  |  |  |  |  |
| **MS & ON history vs. healthy control** |  |  |  |  |  |  |  |  |  |
| MS and ON history |  |  |  |  |  |  |  |  |  |
| Healthy control | (reference) |  |  |  |  |  |  |  |  |
| MS + ON | -22.8 | (-34.3, -11.3) | 0.000 | -27.1 | (-36.2, -18) | 0.000 | -19.4 | (-29.1, -9.6) | 0.000 |
| MS, no ON | -36.2 | (-53.3, -19.2) | 0.000 | -33.6 | (-47.2, -20.1) | 0.000 | -24.7 | (-39.2, -10.2) | 0.001 |
| Age (centered) | -0.9 | (-1.1, -0.7) | 0.000 | -0.6 | (-0.7, -0.4) | 0.000 | -0.4 | (-0.5, -0.2) | 0.000 |
| logMAR | -0.5 | (-26.1, 25) | 0.968 | -1.1 | (-21.4, 19.2) | 0.914 | -3 | (-24.7, 18.6) | 0.784 |
| Pseudophakic | -3.6 | (-13.8, 6.6) | 0.488 | -11.5 | (-19.6, -3.4) | 0.006 | -15.9 | (-24.6, -7.3) | 0.000 |
|  |  |  |  |  |  |  |  |  |  |
|  |  |  |  |  |  |  |  |  |  |
| **MS & RNFL T vs. healthy control** |  |  |  |  |  |  |  |  |  |
| MS & RNFL |  |  |  |  |  |  |  |  |  |
| Healthy control | (reference) |  |  |  |  |  |  |  |  |
| MS + T normal | -22.7 | (-35.1, -10.3) | 0.000 | -19.3 | (-28.9, -9.7) | 0.000 | -10.6 | (-20.8, -0.3) | 0.044 |
| MS + T abnormal | -32.7 | (-47.4, -18) | 0.000 | -43.2 | (-54.6, -31.9) | 0.000 | -36 | (-48.2, -23.9) | 0.000 |
| Age (centered) | -0.9 | (-1.1, -0.7) | 0.000 | -0.6 | (-0.7, -0.4) | 0.000 | -0.4 | (-0.6, -0.2) | 0.000 |
| logMAR | 1.7 | (-24.0, 27.4) | 0.897 | 2.9 | (-17.0, 22.8) | 0.778 | 1.1 | (-20.2, 22.4) | 0.919 |
| Pseudophakic | -3.9 | (-14.1, 6.3) | 0.456 | -12.1 | (-20, -4.2) | 0.003 | -16.5 | (-25, -8.1) | 0.000 |
|  |  |  |  |  |  |  |  |  |  |
| **MS & RNFL G vs. healthy control** |  |  |  |  |  |  |  |  |  |
| MS & RNFL |  |  |  |  |  |  |  |  |  |
| Healthy control | (reference) |  |  |  |  |  |  |  |  |
| MS + G normal | -15.8 | (-28.5, -3.1) | 0.016 | -16.7 | (-26.6, -6.9) | 0.001 | -7.6 | (-18.1, 2.9) | 0.159 |
| MS + G abnormal | -40.6 | (-54.7, -26.5) | 0.000 | -44.5 | (-55.5, -33.6) | 0.000 | -37.8 | (-49.4, -26.1) | 0.000 |
| Age (centered) | -0.9 | (-1.1, -0.7) | 0.000 | -0.6 | (-0.8, -0.4) | 0.000 | -0.4 | (-0.6, -0.2) | 0.000 |
| logMAR | -2.1 | (-27.4, 23.2) | 0.873 | -3.3 | (-22.9, 16.4) | 0.744 | -5.5 | (-26.5, 15.5) | 0.609 |
| Pseudophakic | -1.4 | (-11.6, 8.8) | 0.785 | -9 | (-17, -1.1) | 0.027 | -13.2 | (-21.7, -4.7) | 0.003 |
